# PRINCIPLE trial demonstrates scope for in-pandemic improvement in primary care antibiotic stewardship: a retrospective sentinel network cohort study

**DOI:** 10.3399/BJGPO.2021.0087

**Published:** 2021-09-29

**Authors:** Simon de Lusignan, Mark Joy, Julian Sherlock, Manasa Tripathy, Oliver van Hecke, Kome Gbinigie, John Williams, Christopher Butler, FD Richard Hobbs

**Affiliations:** 1 Professor of Primary Care & Clinical Informatics, Nuffield Department of Primary Care Health Sciences, University of Oxford, Oxford, UK; 2 Senior Researcher, Nuffield Department of Primary Care Health Sciences, University of Oxford, Oxford, UK; 3 SQL Developer, Nuffield Department of Primary Care Health Sciences, University of Oxford, Oxford, UK; 4 Research Officer/Practice Liaison Officer, Nuffield Department of Primary Care Health Sciences, University of Oxford, Oxford, UK; 5 NIHR Academic Clinical Lecturer, Nuffield Department of Primary Care Health Sciences, University of Oxford, Oxford, UK; 6 GP and DPhil Student, Nuffield Department of Primary Care Health Sciences, University of Oxford, Oxford, UK; 7 Senior Research Fellow, Nuffield Department of Primary Care Health Sciences, University of Oxford, Oxford, UK; 8 Professor of Primary Care, Nuffield Department of Primary Care Health Sciences, University of Oxford, Oxford, UK; 9 Head of Department, Nuffield Department of Primary Care Health Sciences, University of Oxford, Oxford, UK

**Keywords:** primary health care, COVID-19, clinical trial, pandemics, medical records systems, computerized, antimicrobial stewardship

## Abstract

**Background:**

The Platform Randomised trial of INterventions against COVID-19 In older peoPLE (PRINCIPLE) has provided in-pandemic evidence that azithromycin and doxycycline were not beneficial in the early primary care management of coronavirus 2019 disease (COVID-19).

**Aim:**

To explore the extent of in-pandemic azithromycin and doxycycline use, and the scope for trial findings impacting on practice.

**Design & setting:**

Crude rates of prescribing and respiratory tract infections (RTI) in 2020 were compared with 2019, using the Oxford Royal College of General Practitioners (RCGP) Research and Surveillance Centre (RSC).

**Method:**

Negative binomial models were used to compare azithromycin and doxycycline prescribing, lower respiratory tract infections (LRTI), upper respiratory tract infections (URTI), and influenza-like illness (ILI) in 2020 with 2019; reporting incident rate ratios (IRR) between years, and 95% confidence intervals (95% CI).

**Results:**

Azithromycin prescriptions increased 7% in 2020 compared with 2019, whereas doxycycline decreased by 7%. Concurrently, LRTI and URTI incidence fell by over half (58.3% and 54.4%, respectively) while ILI rose slightly (6.4%). The overall percentage of RTI-prescribed azithromycin rose from 0.51% in 2019 to 0.72% in 2020 (risk difference 0.214%; 95% CI = 0.211 to 0.217); doxycycline rose from 11.86% in 2019 to 15.79% in 2020 (risk difference 3.93%; 95% CI = 3.73 to 4.14). The adjusted IRR showed azithromycin prescribing was 22% higher in 2020 (IRR = 1.22; 95% CI = 1.19 to 1.26; *P*<0.0001). For every unit rise in confirmed COVID-19 there was an associated 3% rise in prescription (IRR = 1.03; 95% CI = 1.02 to 1.03; *P*<0.0001); whereas these measures were static for doxycycline.

**Conclusion:**

PRINCIPLE demonstrates scope for improved antimicrobial stewardship during a pandemic.

## How this fits in

Antimicrobial stewardship is key to appropriate clinical management of patients and preventing an increase in antimicrobial resistance. With the slowing development of antimicrobials, there is a need to reduce unnecessary prescription to patients who may not benefit from them.

## Introduction

The UK, primary care, prospective adaptive Platform Randomised trial of INterventions against COVID-19 In older peoPLE (PRINCIPLE)^1^ has reported that two antibiotics, azithromycin and doxycycline, show no meaningful benefit in patient-reported recovery for coronavirus 2019 disease (COVID-19).^2,3^ Azithromycin was included in PRINCIPLE between 23 May and 30 November 2020; doxycycline between 24 July and 14 December.

The National Institute for Health and Care Excellence (NICE) states: '*As COVID–19 pneumonia is caused by a virus, antibiotics are ineffective*.' Although at the time of the study, this statement was qualified by the suggestion that where antibiotics were used, doxycycline should be the first choice.^4^ However, GPs may have had a lower threshold for prescribing antibiotics with more remote consultations, the excess COVID-19 associated mortality,^5^ and its associated disparities.^6^

This study was carried out to assess whether there was scope for the PRINCIPLE findings to change practice.

## Method

### Data source and population

Data were used from the Oxford RCGP RSC database, which is an established primary care sentinel network.^7,8^

A convenience sample was included of 397 general practices, with a total registered list size of 4 453 626, where antibiotic drug codes were curated.

### Reporting crude differences

Monthly prescribing rates of azithromycin and doxycycline were reported, comparing 2020 with 2019, also reporting consultations for incident RTI across a period where a range of non-pharmaceutical interventions (NPIs) were implemented.^9,10^ RTIs were subdivided into LRTI, URTI, ILI, and people with COVID-19. Only included incident cases with two or more weeks between events were included.

### Modelling the difference

Negative binomial models were created to report any difference between the use of azithromycin and doxycycline in 2020, compared with 2019. This model was preferred as there was overdispersion in the data. Population at-risk denominators were included in all regression models. The following were adjusted for: age band; sex; socioeconomic status, using the Index of Multiple Deprivation (IMD, derived from post code); NHS region (the Midlands and east regions combined to provide a more balanced distribution); and the incidence of LRTI, URTI, and ILI. Antibiotic use was examined across all age bands, and IRR with 95% CI and significance were reported. People aged ≥65 years were reported separately.

### Sensitivity analysis

A sensitivity analysis was conducted and it was explored whether the RSC practices had higher rates of prescription of antibiotics than the rest of England, as the PRINCIPLE trial participation may have encouraged increased prescribing. National data were used from OpenPrescribing,^11^ using NHS Digital’s national list-size as denominator.^12^ OpenPrescribing data were compared for the first 10 months of the year, as only data to October 2020 were available.

## Results

### Crude rates of azithromycin and doxycycline prescribing

Azithromycin prescriptions increased by 6.98% between 2019 and 2020, while those of doxycycline fell by 7.02% (Supplementary file, Table S4.1).

**Figure 1.**
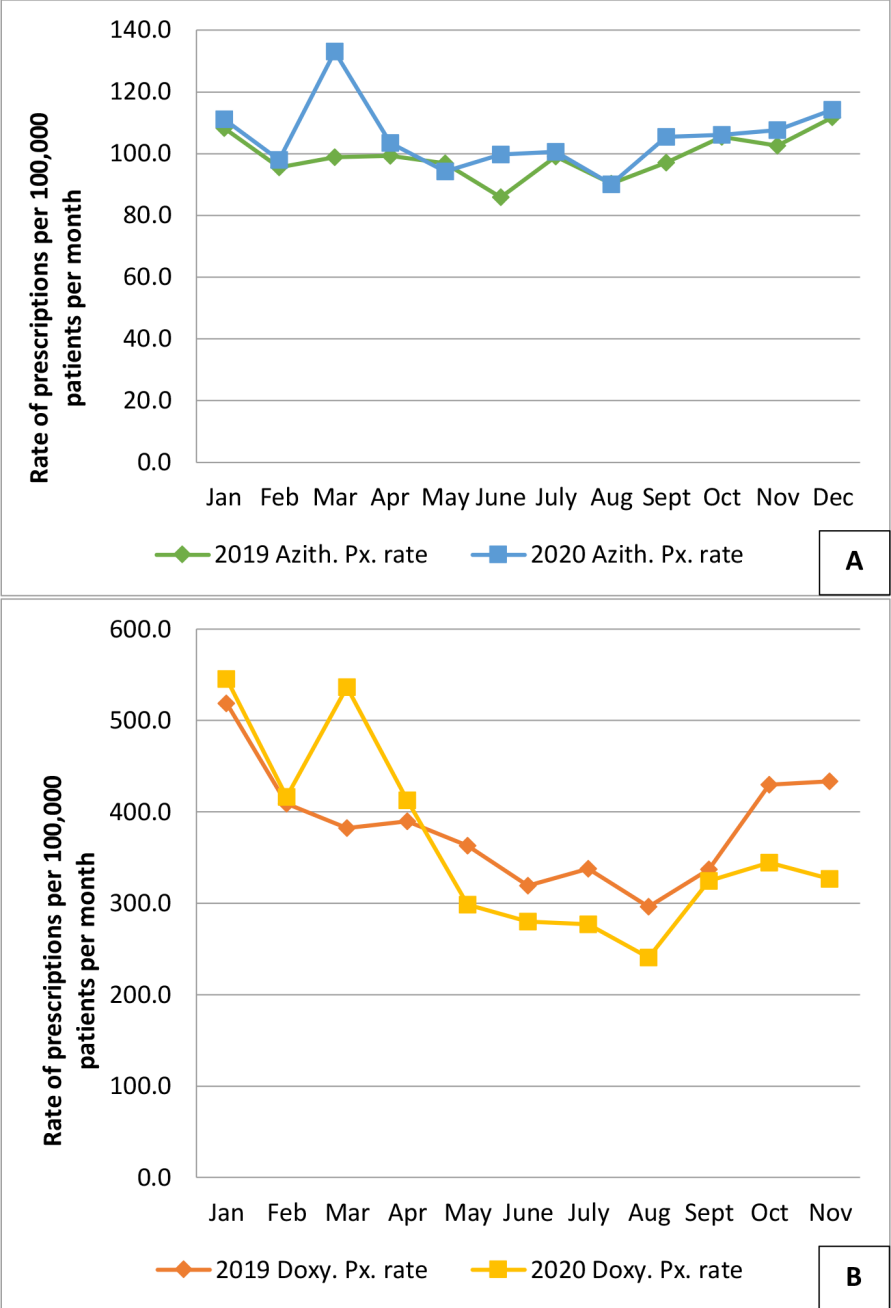
Prescribing of azithromycin (Figure 1A) and doxycycline (Figure 1B) by month within the Research and Surveillance Centre. The 2020 prescription of both antibiotics (azithromycin = blue line, square markers; doxycycline = yellow line, square markers) was very similar in January and February to 2019 rates (azithromycin = green line, diamond markers; doxycycline = orange line; diamond markers). In March 2020, there was a peak of prescribing for both antibiotcs, coincident with the first wave of the COVID-19 pandemic. Thereafter azithromycin was prescribed in 2020 at or above the level in 2019, whereas doxycycline was prescribed less. Azith = azithromycin. Doxy = doxycycline. Px = prescribing.

In January/February 2020, prescriptions of azithromycin and doxycycline were similar to those in 2019. However, in March 2020, prescribing of both antibiotics peaked, coincident with the first wave of COVID-19. Azithromycin was prescribed in 2020 at or above the level prescribed in 2019, whereas the converse was true for doxycycline (Figure 1, Supplementary file S1.1 and S1.2).

**Figure 2.**
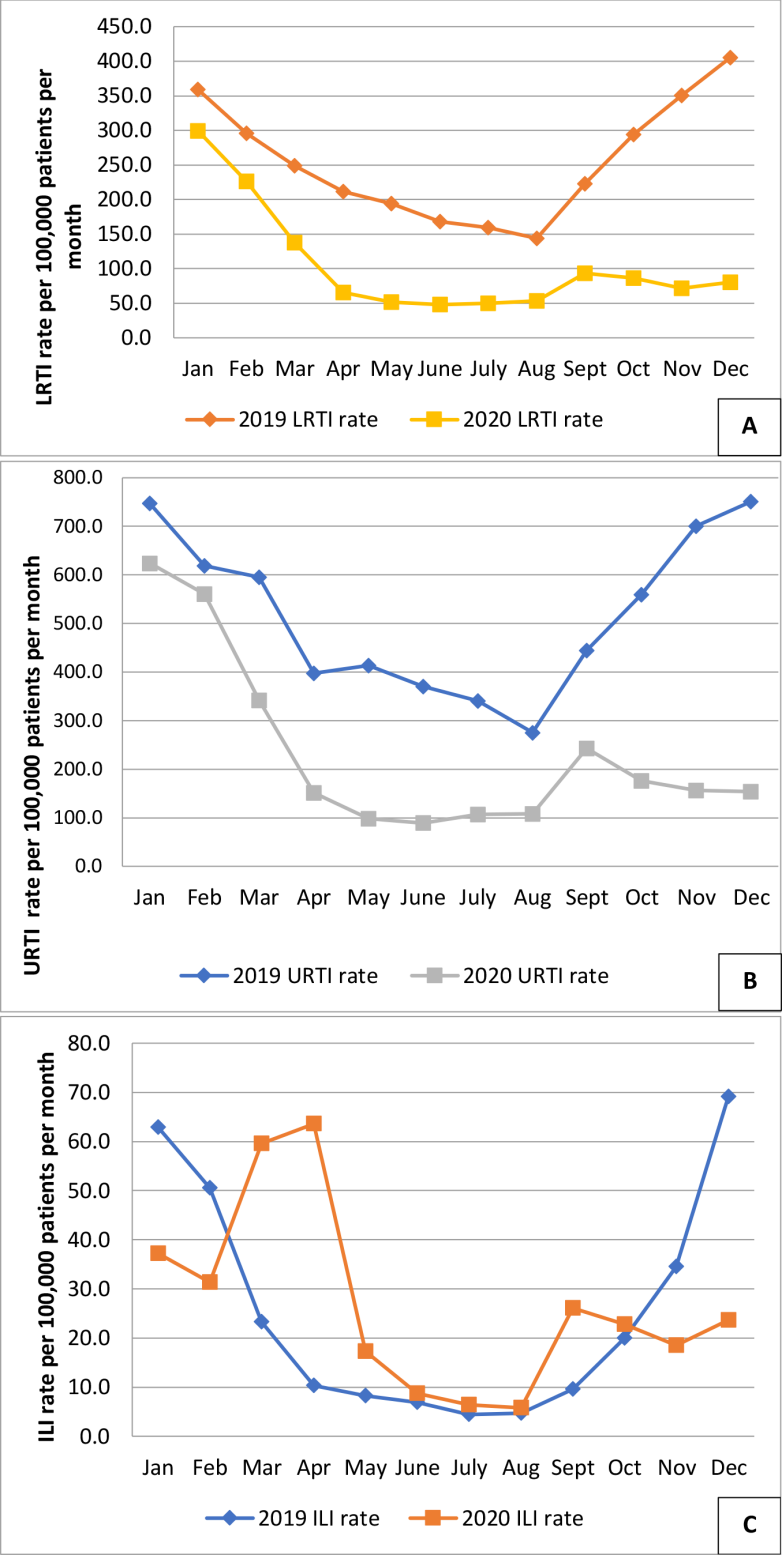
Comparison of monthly incidence of consultations for lower respiratory tract infections (Figure 2A), upper respiratory tract infections (Figure 2B), and influenza-like illness (Figure 2C), comparing 2020 with 2019 in the Research and Surveillance Centre dataset. There was a lower incidence of LRTI and URI in 2020 compared with 2019, with a small peak when pupils returned to school in September 2020. ILI peaked with the first wave of the COVID-19 pandemic, and also with the return to school. ILI = influenza-like illness. RTI = lower respiratory tract infections. URTI = upper respiratory tract infections.

Consultations for LRTI and URTI were over 50% lower in 2020 than 2019. Incidence was lower in every month (Figure 2). ILI incidence in 2019 followed a typical higher winter incidence, whereas 2020 showed peaks that reflected the waves of COVID-19 (Figure 2, Supplementary file S2.3). However, ILI incidence in males aged <16 years fell, while that of females aged 16–64 years was higher (Table 1).

**Table 1.** Comparison of rates of prescription of doxycycline and azithromycin in 2020 with 2019. In people aged ≥65 years there was a decrease in doxycycline use but an increase in azithromycin prescription. Lower respiratory tract infection and upper respiratory tract infection incidence fell across all age bands and both sexes. Influenza-like illness was much more similar between years.

	**Age, years**	2019	2020
Female	Male	Female	Male
**Antibiotic rates per** **100000 patients (95% CI**)	**Doxy**.	<16	21.74(20.4 to 23.2)	17.75(16.5 to 19.0)	19.12(17.8 to 20.5)	15.96(14.8 to 17.2)
		16–64	385.28(382.1 to 388.5)	231.99(229.6 to 234.4)	380.15(377.8 to 383.3)	222.57(220.2 to 224.9)
		≥65	1136.15(1126.4 to 1145.9)	1038.78(1028.6 to 1048.9)	968.28(959.3 to 977.3)	913.57(904.1 to 923.1)
	**Azith**.	<16	56.89(54.6 to 59.2)	67.50(65.1 to 69.9)	53.29(51.1 to 55.5)	75.91(73.4 to 78.5)
		16–64	70.02(68.7 to 71.4)	39.39(38.4 to 40.4)	70.69(69.4 to 72.1)	40.77(39.8 to 41.8)
		≥65	305.17(300.1 to 310.3)	288.43(283.1 to 293.9)	339.13(333.8 to 344.5)	307.59(302.1 to 313.2)
**Respiratory disease rates** **per 100000 patients** **(95% CI**)	**LRTI**	<16	229.25(224.67 to 233.82)	292.18(287.1 to 297.2)	68.43(65.9 to 70.9)	90.9(88.1 to 93.8)
		16–64	191.30(189.1 to 193.54)	126.84(125.0 to 128.6)	81.45(79.9 to 82.9)	53.2(52.0 to 54.4)
		≥65	609.27(602.05 to 616.5)	568.32(560.8 to 575.8)	267.9(263.2 to 272.8)	268.0(262.9 to 273.2)
	**URTI**	<16	1320.33(1309.5 to 1331.3)	1349.86(1339.1 to 1360.7)	485.15(455.0 to 467.7)	493.1(486.6 to 499.7)
		16–64	485.65(482.1 to 489.2)	229.3(226.9 to 231.8)	265.73(254.9 to 260.1)	117.38(115.7 to 119.1)
		≥65	285.17(280.3 to 290.1)	208.6(204.1 to 213.2)	148.01(144.5 to 151.6)	104.61(101.4 to 107.9)
	**ILI**	<16	19.30(17.1 to 19.7)	20.78(19.5 to 22.2)	16.38(15.2 to 17.7)	16.23(15.1 to 17.5)
		16–64	32.57(30.8 to 32.6)	22.15(21.4 to 22.9)	37.68(36.7 to 38.7)	21.86(21.1 to 22.6)
		≥65	25.40(28.2 to 31.7)	22.18(20.7 to 23.7)	29.41(27.9 to 31.0)	24.38(22.9 to 25.9)

^a^95% confidence intervals. Azith = azithromycin. Doxy = doxycycline. ILI = influenza-like illness. LRTI = lower respiratory tract infections. URTI = upper respiratory tract infections.

In 2020, compared with 2019, azithromycin and doxycycline prescribing in RTIs rose by 0.21% (95% CI = 0.211 to 0.217, *P*<0.0001) and 4% (95%CI = 3.73 to 4.14, *P*<0.0001), respectively.

### Modelling the difference

After adjusting for age, sex, socioeconomic status, NHS region, and RTIs, the frequency of azithromycin prescriptions (for any reason) was 22% higher in 2020 compared with 2019 (IRR =1.22; 95% CI = 1.19 to 1.26; *P*<0.0001, Table 2).

**Table 2.** Model reporting the incident rate ratio (IRR) comparing prescribing of azithromycin in 2020 with 2019. Taking the variables in the model into account there was a 22% increase, with people aged ≥65 years, female sex, the most deprived, northern regions and people with lower respiratory tract infections and upper respiratory tract infections all being associated with a higher rate of prescribing.

**Azithromycin prescribing rates**comparing 2020 with 2019	IRR	Lower	Upper	*P*
	95% CI	95% CI
Year 2020 (reference level: 2019)	1.22	1.19	1.26	<0.0001
Age band (reference level: 0–15)				
16–64	0.71	0.68	0.73	<0.0001
*≥* *65*	4.77	4.58	4.98	<0.0001
Sex (reference level: F)	0.91	0.88	0.93	<0.0001
IMD quintile (reference level: Q1, most deprived)				
Q2	0.90	0.86	0.94	<0.0001
Q3	0.87	0.83	0.90	<0.0001
Q4	0.75	0.72	0.78	<0.0001
Q5 (least deprived)	0.67	0.64	0.70	<0.0001
NHS region (reference: London)				
*The* *Midlands and East*	1.08	1.03	1.12	<0.0001
North East and Yorkshire	1.47	1.40	1.54	<0.0001
North West	1.13	1.08	1.18	<0.0001
South East	0.94	0.89	0.98	<0.0001
South West	0.72	0.69	0.76	<0.0001
Respiratory disease				
LRTI count	1.0051	1.0043	1.0058	<0.0001
URTI count	1.0030	1.0026	1.0035	<0.0001
ILI count	1.0017	0.9982	1.0053	0.3400

ILI = influenza-like illness. IMD = Index of Multiple Deprivation. LRTI = lower respiratory tract infections. URTI = upper respiratory tract infections.

For every unit rise in COVID-19 confirmed count there was an associated 3% rise in azithromycin prescription (IRR = 1.03; 95% CI = 1.02 to 1.03; *P*<0.0001, Table 3). With azithromycin, there was a much higher rate of prescribing to those aged ≥65 years, and a lower rate to those aged 16–64 years. There was less azithromycin prescribing for males compared with females, and higher rates of prescribing to the most deprived regions and in the North compared with the South. Comparing 2020 with 2019 overall, there was more azithromycin prescribing for people with LRTI and URTI.

**Table 3.** Azithromycin prescribing in cases of COVID-19. For each unit rise in COVID-19 cases there has been a 3% rise in azithromycin prescriptions. Aged ≥65 years, female sex, being more deprived, northern regions, lower respiratory tract infections or influenza-like-illness infections are all associated with a higher rate of prescribing.

**Azithromycin prescribing rate**	IRR	Lower	Upper	*P*
95% CI	95% CI
COVID-19 confirmed count	1.03	1.02	1.03	<0.0001
Age band (reference level: 0–15)				
16–64	0.25	0.20	0.31	<0.0001
≥65	10.95	8.67	13.83	<0.0001
Sex (reference level: F)	0.54	0.45	0.65	<0.0001
IMD quintile (reference level: Q1 most deprived)				
Q2	0.54	0.41	0.72	<0.0001
Q3	0.41	0.31	0.55	<0.0001
Q4	0.54	0.41	0.72	<0.0001
Q5 (least deprived)	0.66	0.50	0.88	0.0048
NHS region (reference: London)				
The Midlands and East	5.73	4.28	7.69	<0.0001
North East and Yorkshire	12.88	9.18	18.07	<0.0001
North West	10.31	7.34	14.49	<0.0001
South East	2.82	2.01	3.96	<0.0001
South West	1.41	1.01	1.98	0.0453
Respiratory disease				
LRTI count	1.94	1.92	1.97	<0.0001
URTI count	0.89	0.88	0.90	<0.0001
ILI count	1.60	1.54	1.68	<0.0001

ILI = influenza-like-illness. IMD = Index of Multiple Deprivation. LRTI = lower respiratory tract infections. URTI = upper respiratory tract infections.

The same negative binomial model found no change in the rate of prescribing of doxycycline, in 2020 compared with 2019 (IRR = 1.012; 95% CI = 0.994 to 1.030; *P* = 0.199). Female sex, the most deprived quintile, the Midlands and Southwest region, LRTI, and ILI were associated with higher rates of prescription (Supplementary table S3.6).

Adjusting for age, sex, socioeconomic status, region, and RTI, there was a very small rise of 0.3% in the rate of prescribing in doxycycline (IRR = 1.0003; 95% CI = 1.0002 to 1.0005; *P*<0.0001). Female sex, the most deprived quintile, the Midlands and Southwest region, LRTI, and ILI were associated with higher rates of prescription of doxycycline (Supplementary table S3.7).

### Sensitivity analysis

**Figure 3.**
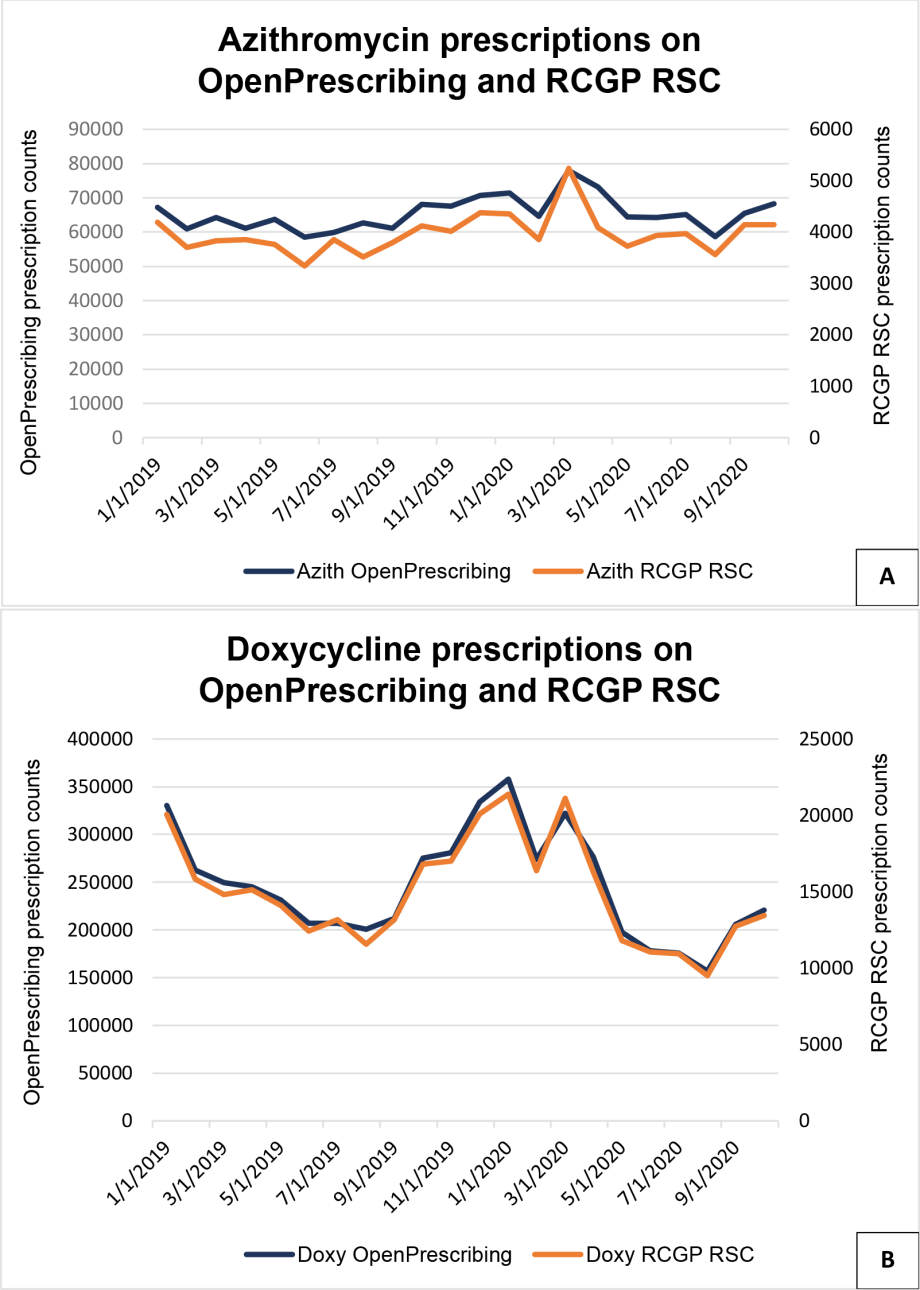
Monthly pattern of azithromycin (Figure 3A) and doxycycline (Figure 3B) prescription counts, for 2019 and 2020. OpenPrescribing data are only available up to October 2020. Azith = azithromycin. Doxy = doxycycline. RCGP RSC = Royal College of General Practitioners Research and Surveillance Centre.

OpenPrescribing showed a very similar pattern of prescribing (Figure 3, Supplementary Table S4.1).

## Discussion

### Summary

Crude rates of azithromycin prescribing increased by 7% in 2020 compared with 2019, while doxycycline prescribing reduced by the same amount (7%).

Prescribing of both antibiotics peaked in the first wave of COVID-19 (March 2020). There was no equivalent peak of prescribing in the second wave. Azithromycin prescribing in 2020 mirrored that of 2019, while doxycycline prescribing in 2020 decreased compared with 2019.

While the incidence of URTI and LRTI was reduced in 2020, ILI increased at the start of the year with circulating influenza, and subsequently mirroring COVID-19 incidence.

The adjusted rate of doxycycline did not change, whereas azithromycin prescribing increased by 22% in 2020 compared with 2019 and as the number of COVID-19 cases increased, azithromycin prescribing increased.

### Strengths and limitations

The PRINCIPLE trial has provided a robust in-pandemic trials platform.^2,3,13^ The strength of this analysis is that the RSC has good data quality and is able to capture routine data about RTIs and their incidence.^14,15^

Comparing the use of azithromycin and doxycycline between years, and their use in RTIs, is complex and the authors are reporting relative change in antibiotic use. Their absolute level of use in RTIs is very low. Both antibiotics have had a significantly increased use in RTIs in 2020 (16.5%) compared with 2019 (12.4%). The decrease in doxycycline is discordant with NICE guidance, which suggested using doxycycline first line.^4^

Additionally, trial drugs may not have been recorded in the GP computer system, as supplied by the clinical trials unit.

### Comparison with existing literature

It is not known why azithromycin prescribing increased during 2020, as estimates of bacterial super-infection are low, at around 3.5%.^16,17^ There were widely reported studies about its use in COVID-19, although these ultimately reported negative outcomes;^18–20^ and remote consultations increased substantially, possibly reducing the threshold for prescribing.^21^

### Implications for practice

The PRINCIPLE trial demonstrated no benefit from either antibiotic in the early treatment of COVID-19 and RSC involvement did not seem to be associated with higher rates of prescribing than those seen in OpenPrescribing.

In conclusion, the PRINCIPLE trial demonstrated the lack of efficacy of azithromycin and doxycycline in primary care. Clinicians should apply good antibiotic stewardship and reduce their use, as these antibiotics are being prescribed in a higher proportion of people with respiratory infections than in the pre-pandemic year. There is scope during the pandemic to reduce the use of azithromycin and doxycycline in primary care.
